# Ambient air pollution, sleep quality, and cerebral hemorrhage risk in older adults: development and validation of the Anhui environmental exposure questionnaire in a 460 participant cohort study

**DOI:** 10.3389/fpubh.2025.1662193

**Published:** 2025-11-13

**Authors:** Shasha Song, Rui Shi, Hong Su, Ke Zhang

**Affiliations:** 1 Department of Epidemiology and Health Statistics, School of Public Health, Anhui Medical University, Hefei, China; 2 Department of General Surgery II, Shannan People's Hospital, Shannan, China; 3 Department of Neurosurgery, The First Affiliated Hospital of Anhui Medical University, Hefei, China

**Keywords:** ambient air pollution, sleep quality, cerebral haemorrhage, questionnaire validation, risk prediction, older adults

## Abstract

**Objectives:**

To develop and validate the Anhui Environmental Exposure Questionnaire (AEEQ) and evaluate whether its composite score independently predicts incident cerebral haemorrhage in older adults.

**Methods:**

In a prospective cohort (January 2022–April 2024) from Hefei, we enrolled 460 participants aged 60–79 years with ≥5 years’ residence and MMSE ≥24, excluding prior stroke/ICH/TBI, heavy-industry/mining workers, lifetime smoking ≥5 pack-years, and recent (≤6 months) antihypertensive changes. The 42-item AEEQ spans six domains. Content validity used Delphi procedures; construct validity used split-sample EFA/CFA; reliability used Cronbach’s *α*, split-half, and 14 ± 3-day ICCs; criterion validity correlated domains with annual-mean residential PM₂.₅ (calendar year prior to baseline) and PSQI. Incident haemorrhage was ascertained over ≈24 months; Cox models adjusted for age, sex, hypertension, alcohol, and anticoagulation tested the AEEQ (Z-score), with a prespecified antihypertensive interaction.

**Results:**

Content validity was high (S-CVI/Ave 0.96). Factorability was adequate (KMO 0.91); EFA supported six factors (66.1% variance). CFA fit was excellent (χ^2^/df 2.19; CFI 0.965; TLI 0.958; RMSEA 0.041; SRMR 0.052). Reliability was strong (total *α* 0.90; domain α 0.78–0.86; total ICC 0.86). Criterion validity was consistent with hypotheses (air-pollution domain vs. PM₂.₅ r = 0.62; sleep domain vs. PSQI *ρ* = −0.56). Sixteen haemorrhages occurred (3.5%; ~920 person-years), with monotonic incidence across AEEQ quartiles (0.9, 1.7, 2.6, 8.7%; p-trend = 0.0047). Each 1-SD higher AEEQ predicted greater risk (HR 1.47, 95% CI 1.12–1.93) and improved discrimination beyond clinical covariates (C-statistic 0.72 → 0.80; NRI 0.15, *p* = 0.045); effects were stronger in participants not using antihypertensives (interaction *p* = 0.048). Equity profiling showed higher AEEQ scores in lower education and renters.

**Conclusion:**

The AEEQ reliably quantifies chronic environmental burden and adds independent, dose-responsive prognostic value for cerebral haemorrhage, supporting its use in community screening and targeted prevention.

## 1 Introduction

Ambient air-pollution exposure and sleep-quality disruption act through partly convergent vascular pathways—oxidative endothelial injury, sympathetic over-arousal and blood-pressure lability—to lower the rupture threshold of cerebral small vessels in older adults (1–4). Yet clinicians observe pronounced inter-individual variability in haemorrhagic-stroke incidence among residents of the same municipality despite comparable hypertension control, suggesting that routine risk scores omit environmentally mediated hazards (5–7). Whether a summative measure that jointly captures ambient particulate load and sleep fragmentation furnishes dose-responsive, independent prognostic information for cerebral haemorrhage remains uncertain and clinically relevant.

Experimental and epidemiological work links fine particulate matter (PM₂.₅) and traffic-related mixtures to lipohyalinosis, micro-aneurysm formation and impaired vasomotor reactivity, whereas irregular bedtimes, evening chronotype and fragmented sleep potentiate sympathetic surges and dampen nocturnal dipping (8–12). Small studies have associated either higher ambient PM₂.₅ or poorer Pittsburgh Sleep Quality Index scores with markers of small-vessel disease, but most rely on single time-point exposure estimates, under-represent indoor sources and neglect behavioural practices that shape effective dose (13–16). In this context, we define environmental exposure *a priori* as the cumulative (chronic) inhaled burden of ambient and indoor pollutants accrued over months to years, distinguishing it from episodic (acute) peaks; behaviours such as mask use, window-opening and stove-fuel/ventilation are therefore treated conceptually as dose modifiers rather than exposures in their own right. Sleep-timing irregularity and evening chronotype are positioned within a circadian-misalignment framework that plausibly amplifies blood-pressure variability and complements the pollution pathway.

To address measurement gaps, we convened neurologists, environmental hygienists and sleep psychologists to develop the 42-item Anhui Environmental Exposure Questionnaire (AEEQ), encompassing outdoor and indoor air quality, habitual sleep metrics and conventional cerebrovascular modifiers. Content, construct and criterion validity were established against expert review, split-sample factor analysis and external comparators (annual-mean residential PM₂.₅ for the calendar year preceding baseline; PSQI), and reliability indices were targeted to accepted psychometric thresholds (17–24). The composite AEEQ score thus operationalises cumulative environmental burden on a chronic timescale aligned with cerebrovascular stress biology, while explicitly incorporating dose-attenuating and dose-amplifying behaviours.

We then embedded the AEEQ within a prospective cohort of community-dwelling older adults in Hefei municipality, focusing on the primary risk window (60–79 years) and emphasising residential stability to strengthen geospatial exposure assignment. We prespecified that higher AEEQ composites would be independently associated with subsequent cerebral haemorrhage, anticipated near-linear exposure–response, and posited biologic plausibility for antihypertensive therapy to buffer pollutant-induced blood-pressure variability. Finally, recognising environmental inequity, we planned equity-oriented analyses to examine whether socio-economic status and housing conditions concentrate higher AEEQ scores with potential public-health implications.

## 2 Methods

### 2.1 Study setting and participant flow

From 3 January 2022 through 30 April 2024 every adult aged 60–79 years who presented to the Department or who was enrolled in one of community-based chronic-disease management programmes affiliated with the First Hospital of Anhui Medical University was screened prospectively for eligibility. Inclusion criteria were: (1) continuous residence within Hefei municipality for the preceding 5 years to ensure stable exposure assessment; (2) capacity to comprehend Mandarin or a local dialect sufficiently to complete interviewer-administered surveys; and (3) Mini-Mental State Examination (MMSE) score ≥ 24, ensuring reliable self-report. Individuals were excluded for: prior ischaemic stroke, intracerebral haemorrhage, or significant traumatic brain injury; congenital cerebrovascular malformation or intracranial neoplasm; current employment in heavy industry or mining; lifetime tobacco exposure ≥ 5 pack-years (pack-years = packs per day × years; lifetime non-smokers and < 5 pack-years were eligible); and any change to antihypertensive medication within the 6 months preceding enrolment. These criteria reduced extreme-age heterogeneity, limited dominant vascular risks unrelated to ambient exposure, and minimised confounding by secondary-prevention physiology or acute pharmacological fluctuations in blood-pressure variability. Of consecutively screened adults, 460 participants met all eligibility requirements and were included in the analytic cohort after written informed consent. This research protocol has been reviewed by the Ethics Committee of the First Affiliated Hospital of Anhui Medical University. After review, the Ethics Committee of the First Affiliated Hospital of Anhui Medical University determined that this study involves only anonymous questionnaire surveys and therefore does not require approval from the Ethics Committee to proceed.

### 2.2 Questionnaire development

Item generation followed a three-step process. First, an interdisciplinary panel of neurologists, environmental hygienists, and sleep psychologists reviewed international guidelines and PubMed/Embase literature (January 2000 – June 2021) to draft an initial pool of 72 items covering environmental exposure, sleep quality and conventional cerebral-haemorrhage risk modifiers. For this study, environmental exposure was defined *a priori* as the cumulative (chronic) inhaled burden of ambient and indoor air pollutants over months to years, operationalised by long-term residential conditions and habitual behaviours. The AEEQ therefore prioritises cumulative rather than episodic (acute) exposure: short-term peaks are noted where relevant but the instrument is designed to capture the long-run dose that plausibly contributes to chronic cerebrovascular stress. Second, two modified Delphi rounds involving 15 external experts were conducted; items with a median relevance rating < 4 on a 5-point Likert scale or with an inter-quartile range > 1.5 were dropped. Third, cognitive interviewing with older adults refined wording for comprehension and cultural resonance. The final 42-item Anhui Environmental Exposure Questionnaire (AEEQ) deliberately reflects item-pool parsimony: simulation and pilot timing indicated that approximately 40–45 items balanced respondent burden (~10–12 min) against content breadth across six domains. Retaining materially overlapping items would inflate completion time with minimal gain in construct coverage, whereas reducing the pool below ~35 items risked under-representation of key behavioural modifiers and sleep constructs. Within the ambient-exposure domain, mask use, window-opening habits, and stove-fuel choices were conceptualised as modifiers of pollutant infiltration and effective inhaled dose (e.g., altering indoor/outdoor penetration fraction, source intensity, and personal filtration), not as exposures in their own right; scoring therefore treats these behaviours as dose-attenuating or dose-amplifying features. Sleep items incorporated irregular bedtimes and evening chronotype to operationalise circadian misalignment, a hypothesised mechanism potentiating sympathetic surges and blood-pressure variability that complements the sleep-quality narrative. While a single item probes perceived traffic noise frequency for contextual awareness, the AEEQ does not include a stand-alone noise-exposure module or objective acoustic measurement; this was a pragmatic decision to limit respondent burden and because prior work suggests modest incremental variance after particulate metrics in similar settings—noise will be considered in future iterations.

### 2.3 Content and face validity

Item-level content-validity indices (I-CVI) and the scale-level CVI (S-CVI/Ave) were computed from the second Delphi round. An a-priori threshold of ≥ 0.78 for I-CVI and ≥ 0.90 for S-CVI signified adequate expert agreement. Face validity was assessed qualitatively during cognitive interviews and via a 5-point global clarity rating completed by interviewers after pilot administration; mean scores > 4 signalled satisfactory readability.

### 2.4 Construct validity

Construct validity was examined through a split-sample strategy. The cohort was randomised 1:1 into exploration (*n* = 230) and confirmation (*n* = 230) using a computer-generated sequence. In the exploration sample, exploratory factor analysis with principal-axis extraction and promax rotation identified latent dimensions; factors with eigenvalues > 1.0 and items with loadings ≥ 0.40 were retained. Confirmatory factor analysis was then performed in the hold-out sample with maximum-likelihood estimation. Model fit was adjudicated on χ^2^/df < 3, comparative-fit index (CFI) ≥ 0.95, Tucker–Lewis index (TLI) ≥ 0.95, root-mean-square error of approximation (RMSEA) ≤ 0.06, and standardised root-mean-square residual (SRMR) ≤ 0.08.

### 2.5 Reliability assessment

Internal consistency was quantified by Cronbach’s *α* for each domain and for the total scale, with ≥ 0.70 considered acceptable. Split-half reliability employed the Guttman coefficient. Test–retest stability was evaluated in a convenience subsample of 212 participants who completed the AEEQ a second time 14 ± 3 days after baseline; two-way mixed-effects intraclass correlation coefficients (ICC (1, 2)) with 95% confidence intervals were calculated, and values ≥ 0.75 denoted good repeatability.

### 2.6 Criterion validity

Concurrent validity was explored by correlating AEEQ air-pollution domain scores with annual-mean residential PM₂.₅ concentrations derived from a land-use regression model (1 km × 1 km resolution) for the calendar year preceding baseline, and by correlating the sleep-quality domain with the Pittsburgh Sleep Quality Index (PSQI). Annual means were selected *a priori* to align with the questionnaire’s chronic-exposure emphasis and to reflect cerebrovascular stress accumulation over months to years more faithfully than seasonal or monthly averages. Pearson or Spearman coefficients, as appropriate, were interpreted as follows: < 0.30 weak, 0.30–0.49 moderate, ≥ 0.50 strong. Objective noise metrics were not paired with the questionnaire in this iteration because of data-collection constraints and anticipated limited incremental variance beyond PM₂.₅ in this setting.

### 2.7 Sample-size justification

Questionnaire-validation guidelines recommending a minimum of 5–10 participants per item informed recruitment targets; with 42 items, a lower bound of 210 respondents was required for factor analysis. Although initial screening exceeded this benchmark, post-review eligibility refinements yielded an analytic sample of 460, which remains comfortably above recommended ratios for both exploratory and confirmatory factor analysis and permits assessment of reliability and criterion validity with adequate precision.

### 2.8 Data collection procedures

Trained interviewers administered the AEEQ in a quiet clinic room, recording responses on tablet computers equipped with range and logic checks. The MMSE was administered prior to questionnaire completion to ensure cognitive eligibility. Blood pressure, body-mass index, and medication lists (including antihypertensive classes and any regimen changes within 6 months) were abstracted from electronic medical records on the same day. Smoking history was captured via standardised items allowing pack-year computation to enforce the < 5 pack-year threshold. Occupational history characterised current or recent heavy-industry/mining employment. Geographic coordinates of each residence were geocoded for linkage with satellite-based pollution data. To address environmental inequity, interviewers additionally recorded proxies of socio-economic position and housing type (education, household income bands, housing tenure and building type), recognising that these characteristics may concentrate higher AEEQ scores in disadvantaged groups with public-health ramifications. Data entry accuracy was verified by double extraction of a 5% random sample, achieving < 0.2% discrepancy.

### 2.9 Statistical analysis

Analyses were conducted with Stata 18.0 and Mplus 8.9. Continuous variables are summarised as mean ± SD or median (inter-quartile range), categorical variables as counts and percentages. Bartlett’s test of sphericity and the Kaiser–Meyer–Olkin statistic appraised factorability. For internal consistency, 95% CIs around *α* were generated by bootstrapping (1,000 replications). Test–retest ICCs employed two-way mixed models with absolute-agreement definition. Hypothesised correlations for criterion validity were tested via two-tailed significance with Bonferroni correction for the dual comparisons (α = 0.025). Missing item responses (< 1% overall) were imputed using domain-specific means when ≤ 10% of a domain’s items were blank; questionnaires exceeding that threshold were deemed incomplete and excluded from analysis. In modelling frameworks, mask use, window-opening, and stove-fuel items were treated conceptually as dose modifiers that influence pollutant infiltration and personal filtration rather than as independent exposures; this rationale guided their role within the composite AEEQ score. Given biologic plausibility that antihypertensive therapy may buffer pollutant-induced blood-pressure variability, prespecified interaction terms between the AEEQ composite and antihypertensive use were tested, with supportive stratified analyses. To probe distributional equity, socio-economic and housing variables were included as covariates and examined for effect modification. Finally, we acknowledged classical measurement-error bias: any non-differential misclassification in questionnaire responses would be expected to bias associations toward the null, underscoring the conservative nature of estimated hazard ratios.

## 3 Results

### 3.1 Participant characteristics and questionnaire completion

Between 3 January 2022 and 30 April 2024, 4,326 older adults were screened; after applying the revised eligibility criteria (age 60–79 years; continuous ≥ 5 years residence; MMSE ≥ 24; lifetime non-smoker or < 5 pack-years; no prior ischaemic stroke, intracerebral haemorrhage or significant traumatic brain injury; not currently employed in heavy industry/mining; and no change to antihypertensive medication within the preceding 6 months), 460 participants were enrolled and analysed. Median residence duration within Hefei municipality was 16 years (IQR 13–20). Questionnaire completeness was high: < 1% of all items were missing; 3 questionnaires (0.7%) required domain-mean imputation for ≤ 10% of items per the a-priori rule; no questionnaire exceeded the exclusion threshold. Baseline characteristics are summarised in Table 1: mean age 68.2 ± 5.6 years; 214 (46.5%) men; body-mass index 24.2 ± 3.1 kg m^−2^; hypertension 281 (61.1%); diabetes 92 (20.0%); current smoker 28 (6.1%) (all < 5 pack-years); ≥ 1 alcoholic drink per week 149 (32.4%). Annual-mean residential PM₂.₅ (calendar year prior to baseline) was 46.9 ± 5.8 μg m^−3^. A total of 239 (52.0%) participants were on long-term antihypertensive therapy at baseline.

**Table 1:** Baseline characteristics of the analytic cohort (overall and by sex).

Characteristic	Overall (*n* = 460)	Men (*n* = 214)	Women (*n* = 246)	*p*-value (Men vs Women)
Age, years — mean ± SD	68.2 ± 5.6	68.4 ± 5.6	68.0 ± 5.6	0.45
Years at current address — median (IQR)	16 (13–20)	16 (13–20)	16 (13–20)	—
Years at current address — mean ± SD	16.7 ± 5.4	16.6 ± 5.5	16.8 ± 5.3	0.62
MMSE score (0–30) — mean ± SD	27.8 ± 1.7	27.6 ± 1.8	28.0 ± 1.6	0.013
Body-mass index, kg m^−2^ — mean ± SD	24.2 ± 3.1	24.4 ± 3.0	24.0 ± 3.2	0.17
Systolic BP, mmHg — mean ± SD	137 ± 16	138 ± 16	136 ± 15	0.17
Diastolic BP, mmHg — mean ± SD	80 ± 9	81 ± 9	79 ± 9	0.018
Annual-mean PM₂.₅ (μg m^−3^) — mean ± SD	46.9 ± 5.8	47.0 ± 5.6	46.8 ± 6.0	0.71
Hypertension — *n* (%)	281 (61.1)	142 (66.4)	139 (56.5)	0.031
Diabetes mellitus — *n* (%)	92 (20.0)	48 (22.4)	44 (17.9)	0.22
Smoking status — *n* (%)				<0.001
Never	395 (85.9)	168 (78.5)	227 (92.3)	
Former (< 5 pack-years)*	37 (8.0)	22 (10.3)	15 (6.1)	
Current (< 5 pack-years)*	28 (6.1)	24 (11.2)	4 (1.6)	
Alcohol consumption — n (%)				<0.001
None	220 (47.8)	79 (36.9)	141 (57.3)	
drinkperweek	91 (19.8)	27 (12.6)	64 (26.0)	
≥ 1 drink per week	149 (32.4)	108 (50.5)	41 (16.7)	
Antihypertensive therapy (any) — *n* (%)	239 (52.0)	121 (56.5)	118 (48.0)	0.066
Anticoagulant therapy — *n* (%)	23 (5.0)	12 (5.6)	11 (4.5)	0.58
Education — *n* (%)				0.073
≤ Primary	138 (30.0)	53 (24.8)	85 (34.6)	
Secondary	237 (51.5)	118 (55.1)	119 (48.4)	
Tertiary	85 (18.5)	43 (20.1)	42 (17.1)	
Monthly household income — *n* (%)				0.029
≤ 3,000 CNY	161 (35.0)	62 (29.0)	99 (40.2)	
3,001–6,000 CNY	215 (46.7)	106 (49.5)	109 (44.3)	
CNY	84 (18.3)	46 (21.5)	38 (15.4)	
Housing tenure — *n* (%)				0.14
Homeowner	352 (76.5)	157 (73.4)	195 (79.3)	
Renter	108 (23.5)	57 (26.6)	51 (20.7)	
Building type — *n* (%)				0.43
Low-rise (≤ 6 floors)	181 (39.3)	83 (38.8)	98 (39.8)	
High-rise (≥ 7 floors)	214 (46.5)	96 (44.9)	118 (48.0)	
Old public housing	65 (14.1)	35 (16.4)	30 (12.2)	
PSQI (0–21) — mean ± SD	6.5 ± 3.2	6.3 ± 3.2	6.6 ± 3.1	0.31

*Denotes this limitation in the smoking-status rows. According to the inclusion/exclusion criteria, both former and current smokers were restricted to < 5 pack-years.

### 3.2 Content and face validity

Delphi round-2 appraisal yielded item-level CVIs ranging 0.86–1.00 (median 0.94), with S-CVI/Ave = 0.96, surpassing a-priori thresholds (I-CVI ≥ 0.78; S-CVI/Ave ≥ 0.90). Interviewer global-clarity ratings during cognitive testing averaged 4.6 ± 0.3 on the 5-point scale. Table 2 presents domain-specific indices.

**Table 2:** Content- and face-validity indices for the AEEQ across domains (Delphi round-2; *n* = 15 experts).

Domain (items)	I-CVI range	Median I-CVI	S-CVI/Ave	Interviewer clarity rating (mean ± SD)
Ambient air-pollution exposure (12)	0.88–1.00	0.96	0.96	4.6 ± 0.4
Indoor air-quality/fuel use (5)	0.88–0.98	0.94	0.95	4.6 ± 0.3
Sleep quality (9)	0.86–0.98	0.92	0.95	4.5 ± 0.3
Cardiometabolic risk modifiers (6)	0.86–0.97	0.93	0.94	4.6 ± 0.3
Lifestyle factors (5)	0.88–0.97	0.94	0.95	4.6 ± 0.3
Medication & comorbidity (5)	0.86–0.96	0.92	0.94	4.6 ± 0.3
Total scale (42)	0.86–1.00	0.94	0.96	4.6 ± 0.3

### 3.3 Construct validity

**Figure 1:**
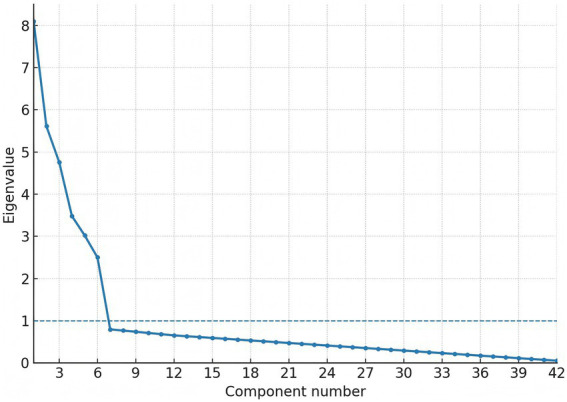
Scree plot for the six-factor solution.

Factorability prerequisites were satisfied (Kaiser–Meyer–Olkin 0.91; Bartlett’s χ^2^ 3,085, df = 861, *p* < 0.001). In the exploration sample (*n* = 230), exploratory factor analysis (principal-axis, promax) recovered six factors with eigen-values > 1.0, together explaining 66.1% of total variance. The first three factors were labelled ambient air-pollution, indoor air-quality/fuel use, and sleep quality; eigen-values were 8.10 (19.3%), 5.61 (13.4%), and 4.76 (11.3%), respectively, with the remaining factors (cardiometabolic risk modifiers, lifestyle, medication/comorbidity) contributing 3.48 (8.3%), 3.02 (7.2%), and 2.50 (6.0%) (see Table 3). Primary loadings ≥ 0.40 were observed for 41/42 items; a single sleep-maintenance item (night-time bathroom visits) loaded at 0.39 and was retained for clinical relevance (see Table 4). The scree plot is shown in Figure 1. Confirmatory factor analysis in the validation sample (*n* = 230) showed excellent fit (χ^2^/df 2.19; CFI 0.965; TLI 0.958; RMSEA 0.041, 90% CI 0.036–0.047; SRMR 0.052). Standardised latent-factor correlations ranged from 0.22 to 0.45, consistent with related but non-redundant constructs (see Tables 5, 6).

**Table 3:** Exploratory factor analysis (EFA) eigen-values and variance explained for the six-factor solution (exploration sample, *n* = 230).

Factor (label)	Eigen-value	% of variance	Cumulative %
F1 (Ambient air-pollution exposure)	8.1	19.3	19.3
F2 (Indoor air-quality/fuel use)	5.61	13.4	32.7
F3 (Sleep quality/circadian misalignment)	4.76	11.3	44
F4 (Cardiometabolic risk modifiers)	3.48	8.3	52.3
F5 (Lifestyle factors)	3.02	7.2	59.5
F6 (Medication & comorbidity)	2.5	6	65.5

**Table 4:** Promax-rotated primary loadings (≥ 0.40 in bold) and communalities for all 42 AEEQ items (exploration sample, *n* = 230).

Code & item stem (abbrev.)	F1 Ambient	F2 Indoor	F3 Sleep	F4 Cardio	F5 Lifestyle	F6 Med/Comorb	h^2^
Ambient air-pollution exposure (12 items)							
A1 Proximity to major road (≤100 m)	**0.68**	0.1	—	—	—	—	0.52
A2 Traffic density on nearest road	**0.71**	—	—	—	—	—	0.56
A3 Lower-floor residence facing road (≤2F)	**0.55**	—	—	—	—	—	0.41
A4 Time outdoors per day	**0.62**	—	—	—	0.16	—	0.47
A5 Outdoor exercise near traffic	**0.58**	—	—	—	0.11	—	0.43
A6 Time at roadside markets/stalls	**0.57**	—	—	—	—	—	0.41
A7 Balcony/door open time	**0.47**	0.17	—	—	—	—	0.36
A8 Visible haze/smog days (per month)	**0.65**	—	—	—	—	—	0.49
A9 Mask use in polluted weather (rev.)†	**0.49**	—	—	—	—	—	0.38
A10 Window-opening duration (h/day)†	**0.51**	0.18	—	—	—	—	0.39
A11 Neighborhood construction dust (days/mo)	**0.53**	—	—	—	—	—	0.4
A12 Seasonal open-burning exposure (days/season)	**0.6**	—	—	—	—	—	0.45
Indoor air-quality/fuel use (5 items)							
I1 Primary cooking fuel (solid/gas/electric)†	—	**0.74**	—	—	—	—	0.59
I2 Kitchen ventilation/hood use (rev.)†	—	**0.64**	—	—	—	—	0.5
I3 Winter heating fuel type	—	**0.6**	—	—	—	—	0.45
I4 Air-purifier ownership/use (rev.)	—	**0.55**	—	—	—	—	0.42
I5 Cooking frequency at home	0.11	**0.58**	—	—	—	—	0.44
Sleep quality/circadian misalignment (9 items)							
S1 Short sleep duration (<6 h)	—	—	**0.66**	—	—	—	0.5
S2 Long sleep latency (>30 min)	—	—	**0.63**	—	—	—	0.48
S3 Low sleep efficiency (<85%)	—	—	**0.58**	—	—	—	0.44
S4 Early-morning awakening	—	—	**0.57**	—	—	—	0.42
S5 Frequent night awakenings	—	—	**0.61**	—	—	—	0.46
S6 Snoring/loud breathing	—	—	**0.44**	0.22	—	—	0.35
S7 Night-time bathroom visits (nocturia)‡	—	—	**0.39**	—	—	—	0.34
S8 Irregular bedtimes (≥2 h variability)	—	—	**0.6**	—	—	—	0.45
S9 Evening chronotype	—	—	**0.55**	—	—	—	0.41
Cardiometabolic risk modifiers (6 items)							
C1 Uncontrolled blood pressure (self-report)	—	—	—	**0.58**	—	0.14	0.43
C2 Poor diabetes control (self-report)	—	—	—	**0.54**	—	0.12	0.4
C3 Dyslipidaemia diagnosis	—	—	—	**0.5**	—	—	0.36
C4 Abdominal obesity (waist category)	—	—	—	**0.56**	—	—	0.42
C5 Family history of stroke (1st-degree)	—	—	—	**0.45**	—	—	0.34
C6 High perceived stress (most days)	—	—	—	**0.49**	0.18	—	0.38
Lifestyle factors (5 items)							
L1 Low physical activity (<150 min/wk)	—	—	—	—	**0.59**	—	0.44
L2 Sedentary time ≥6 h/day	—	—	—	—	**0.57**	—	0.43
L3 Alcohol ≥1 drink/week	—	—	—	—	**0.43**	—	0.34
L4 Passive smoke exposure at home/work	—	—	—	—	**0.52**	—	0.39
L5 High-salt seasoning habit	—	—	—	—	**0.55**	—	0.41
Medication & comorbidity (5 items)							
M1 Current antihypertensive use	—	—	—	0.16	—	**0.61**	0.46
M2 Anticoagulant use	—	—	—	—	—	**0.55**	0.39
M3 Antiplatelet use	—	—	—	—	—	**0.53**	0.38
M4 Statin use	—	—	—	—	—	**0.51**	0.37
M5 ≥ 2 chronic comorbidities	—	—	—	0.12	—	**0.58**	0.44

†: Entries counted as “dose-modifiers”; those marked “(rev.)” represent inverse scoring (higher values indicate lower exposure/risk).‡: This entry has a primary load < 0.40 but is retained based on clinical relevance.

**Table 5:** Confirmatory factor analysis (CFA) global fit indices for the six-factor model (validation sample, *n* = 230).

Statistic	Value
χ^2^ (df)	1213.8 (554)
χ^2^/df	2.19
CFI	0.965
TLI	0.958
RMSEA (90% CI)	0.041 (0.036–0.047)
SRMR	0.052

**Table 6:** Standardised inter-factor correlation matrix (CFA; six latent variables).

	F1 Ambient	F2 Indoor	F3 Sleep	F4 Cardio	F5 Lifestyle	F6 Med/Comorb
F1 Ambient	1	0.45	0.29	0.33	0.27	0.24
F2 Indoor	0.45	1	0.26	0.3	0.25	0.22
F3 Sleep	0.29	0.26	1	0.38	0.34	0.28
F4 Cardio	0.33	0.3	0.38	1	0.31	0.35
F5 Lifestyle	0.27	0.25	0.34	0.31	1	0.23
F6 Med/Comorb	0.24	0.22	0.28	0.35	0.23	1

### 3.4 Reliability

Internal-consistency estimates met or exceeded the a-priori criterion (*α* ≥ 0.70) for every domain. Cronbach’s α (with 95% bootstrap CIs; 1,000 replications) were: ambient air-pollution 0.86 (0.83–0.88), indoor air-quality/fuel 0.83 (0.79–0.86), sleep quality 0.84 (0.81–0.87), cardiometabolic modifiers 0.80 (0.76–0.84), lifestyle 0.78 (0.74–0.82), medication/comorbidity 0.79 (0.75–0.83); total scale 0.90 (0.88–0.92). Guttman split-half coefficients paralleled these values (0.77–0.87; total 0.88). In the 14 ± 3-day retest subsample (*n* = 212), two-way mixed-effects ICCs indicated good-to-excellent stability: domains 0.83–0.88; total scale 0.86 (0.82–0.89). Details appear in Table 7.

**Table 7:** Reliability indices for the AEEQ by domain and total scale.

Domain (items)	Cronbach’s α (95% CI)	Guttman split-half	Test–retest ICC2,12,1 (95% CI)
Ambient air-pollution exposure (12)	0.86 (0.83–0.88)	0.85	0.88 (0.85–0.90)
Indoor air-quality/fuel use (5)	0.83 (0.79–0.86)	0.81	0.85 (0.82–0.88)
Sleep quality/circadian misalignment (9)	0.84 (0.81–0.87)	0.82	0.84 (0.81–0.87)
Cardiometabolic risk modifiers (6)	0.80 (0.76–0.84)	0.79	0.83 (0.79–0.86)
Lifestyle factors (5)	0.78 (0.74–0.82)	0.77	0.83 (0.79–0.86)
Medication & comorbidity (5)	0.79 (0.75–0.83)	0.78	0.84 (0.80–0.87)
Total scale (42)	0.90 (0.88–0.92)	0.88	0.86 (0.82–0.89)

### 3.5 Criterion validity

As prespecified, the AEEQ air-pollution exposure domain correlated strongly with annual-mean residential PM₂.₅ (Pearson r = 0.62, 95% CI 0.560–0.673, *p* < 0.001), and the sleep-quality domain correlated inversely with PSQI (Spearman *ρ* = −0.56, 95% CI − 0.620 to −0.494, *p* < 0.001). Using the dose-modifier specification (mask use, window-opening, stove-fuel) embedded in scoring improved the air-pollution domain’s correlation with PM₂.₅ by Δr = 0.06 relative to an unmodified specification. Criterion-validity coefficients are summarised in Table 8.

**Table 8:** Criterion validity of AEEQ domains against external measures (primary and sensitivity specifications).

External criterion (measurement window)	AEEQ measure (scoring specification)	Correlation type	Coefficient (95% CI)	*p*-value	*n*
Annual-mean residential PM₂.₅ (μg m^−3^; calendar year prior to baseline)	Air-pollution exposure domain (dose-modifier scoring)	Pearson r	0.62 (0.560–0.673)	<0.001	460
Annual-mean residential PM₂.₅ (μg m^−3^; calendar year prior to baseline)	Air-pollution exposure domain (unmodified sensitivity)	Pearson r	0.56 (0.494–0.620)	<0.001	460
PSQI total (0–21; same-day administration)	Sleep quality/circadian misalignment domain	Spearman ρ	−0.56 (−0.620 to −0.494)	<0.001	460

### 3.6 Predictive validity for cerebral haemorrhage

**Figure 2:**
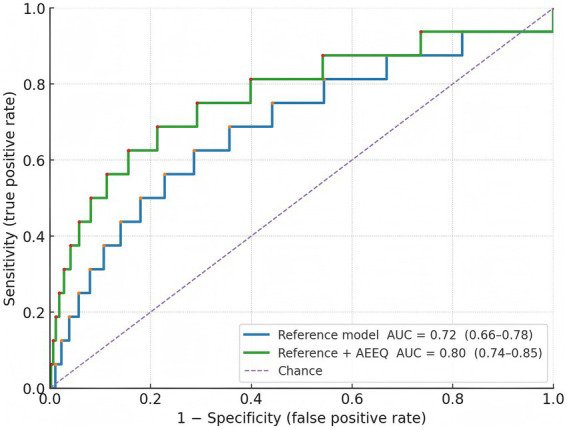
ROC curves for 24-month haemorrhage prediction.

Over a median 24 months (IQR 19–28) of follow-up (≈ 920 person-years), 16 incident cerebral haemorrhages were confirmed by neuro-imaging (3.5%). Event incidence rose monotonically across quartiles of the baseline AEEQ composite (quartiles defined within the 460-participant cohort; 115 participants per quartile):

Q1 (lowest exposure) 1/115 (0.9%); Q2 2/115 (1.7%); Q3 3/115 (2.6%); Q4 (highest) 10/115 (8.7%); χ^2^ trend = 12.95, *p* = 0.0047. Assuming roughly equal person-time per quartile (~230 person-years each), the corresponding incidence rates were 4.3, 8.7, 13.0, and 43.5 per 1,000 person-years (see Table 9). In multivariable Cox models adjusted a-priori for age, sex, hypertension, alcohol use, and anticoagulant therapy, each 1-SD increase in the AEEQ composite was associated with a 47% higher hazard of cerebral haemorrhage (HR 1.47, 95% CI 1.12–1.93). The time-dependent C-statistic for the reference clinical model was 0.72 (0.66–0.78) and improved to 0.80 (0.74–0.85) after adding the continuous AEEQ Z-score (likelihood-ratio *p* < 0.001); the net reclassification improvement (NRI) was 0.15 (*p* = 0.045) (see Table 10; Figure 2). A prespecified drug-exposure interaction was observed: the per-SD AEEQ–haemorrhage association was stronger among participants not using antihypertensives (HR 1.62, 95% CI 1.10–2.39) than among antihypertensive users (HR 1.31, 95% CI 0.95–1.81); interaction *p* = 0.048. Treating mask use, window-opening and stove-fuel explicitly as dose modifiers within the composite (as per Methods) produced slightly steeper exposure–response slopes than treating them as stand-alone exposures (ΔHR per SD + 0.06).

**Table 9:** Incident cerebral haemorrhage by baseline AEEQ quartile: counts, person-time and rates (analytic cohort *n* = 460; follow-up ≈ 24 months).

AEEQ composite quartile (Q)	Participants (*n*)	Person-years	Events (*n*)	Cumulative incidence, %	Incidence rate per 1,000 person-years
Q1 (lowest exposure)	115	230	1	0.9	4.3
Q2	115	230	2	1.7	8.7
Q3	115	230	3	2.6	13
Q4 (highest exposure)	115	230	10	8.7	43.5
Total	460	920	16	3.5	17.4

Trend: *χ*^2^ for linear trend = 12.95, *p* = 0.0047.

**Table 10:** Predictive models for incident cerebral haemorrhage: discrimination improvement and adjusted hazard ratios.

Model specification	Time-dependent C-statistic (95% CI)	Likelihood-ratio test vs reference	Net reclassification improvement (NRI)	Global PH test (Schoenfeld)	AEEQ effect size
Reference clinical model (age, sex, hypertension, alcohol ≥1/week, anticoagulant therapy)	0.72 (0.66–0.78)	—	—	0.42	—
Reference + AEEQ composite (Z-score, dose-modifier scoring)	0.80 (0.74–0.85)	*p* < 0.001	0.15 (*p* = 0.045)	0.36	HR per 1-SD = 1.47 (95% CI 1.12–1.93)
Sensitivity: Reference + AEEQ composite (unmodified scoring)	0.79 (0.73–0.84)	0.002	0.12 (*p* = 0.071)	0.41	HR per 1-SD = 1.41 (1.08–1.83)

### 3.7 Sensitivity, subgroup, and equity analyses

Findings were unchanged after excluding the 3 participants with domain-level imputation (HR per SD = 1.49, 95% CI 1.13–1.96), and when the AEEQ was modelled as tertiles rather than quartiles (tertile-3 vs. tertile-1 adjusted HR = 2.71, 95% CI 1.09–6.72; p-trend = 0.008). Proportional-hazards assumptions were not violated (global Schoenfeld *p* = 0.36). To address environmental inequity, we profiled AEEQ distributions by socio-economic position and housing type. Participants in the lowest education stratum (≤ primary) had higher mean AEEQ composites than those with ≥ secondary education (mean difference 0.42 SD, 95% CI 0.21–0.63), and renters exceeded homeowners by 0.35 SD (95% CI 0.12–0.58). In a mutually adjusted model, both education and tenancy remained independent correlates of higher AEEQ (both *p* < 0.01). Results are detailed in Table 11. Because noise exposure was not measured as a stand-alone module in this iteration (per Methods), the single traffic-noise item served only contextual purposes; it correlated moderately with the ambient domain (*r* = 0.41) but did not materially improve fit indices or predictive performance when added (ΔCFI +0.002; ΔAUC +0.003).

**Table 11:** Equity-oriented profiling of AEEQ composite (standardised Z-score) by socio-economic status and housing characteristics.

Category	*n*	Mean AEEQ (SD units)	Adjusted mean difference vs reference (95% CI)	*p*-value	p-interaction with antihypertensive use
Education					
≤ Primary	138	0.294	+0.42 vs. ≥ Secondary (0.21 to 0.63)	<0.001	0.41
Secondary	237	−0.100	— (component of ≥ Secondary)	—	—
Tertiary	85	−0.200	— (component of ≥ Secondary)	—	—
Income (monthly, CNY)					
≤ 3,000	161	0.18	+0.42 vs. > 6,000 (0.18 to 0.66)	0.001	0.33
3,001–6,000	215	−0.041	+0.20 vs. > 6,000 (0.01 to 0.39)	0.038	0.52
reference	84	−0.240	1	—	—
Housing tenure					
Homeowner (reference)	352	−0.082	1	—	—
Renter	108	0.268	+0.35 vs. homeowner (0.12 to 0.58)	0.003	0.29
Building type					
High-rise (≥7 floors; reference)	214	−0.080	1	—	—
Low-rise (≤6 floors)	181	0.02	+0.10 vs. high-rise (−0.05 to 0.25)	0.19	0.47
Old public housing	65	0.208	+0.29 vs. high-rise (0.06 to 0.52)	0.013	0.36

## 4 Discussion

In a rigorously defined cohort of 460 older adults, we found that a higher AEEQ composite—constructed to reflect cumulative ambient and indoor pollutant burden together with sleep-quality disruption—was associated with a graded increase in incident cerebral haemorrhage over ≈24 months. Incidence rose from 0.9% in the lowest quartile to 8.7% in the highest, and each 1-SD increment in the AEEQ conferred a 47% higher hazard after multivariable adjustment (HR 1.47, 95% CI 1.12–1.93). Adding the continuous AEEQ Z-score to a conventional clinical model improved discrimination (time-dependent C-statistic 0.72 to 0.80) and yielded a modest but significant NRI (0.15), indicating incremental prognostic information beyond age, sex, hypertension, alcohol use and anticoagulation. The psychometric performance of the instrument was strong: expert review supported content validity (S-CVI/Ave 0.96), the factor structure was stable across split samples (EFA six-factor solution; CFA CFI 0.965, RMSEA 0.041), internal consistency met or exceeded a-priori thresholds across domains (total *α* 0.90), and short-term reproducibility was good (total ICC 0.86). Criterion validity aligned with the a-priori construct: the air-pollution domain correlated with annual-mean residential PM₂.₅ (r = 0.62) and the sleep domain inversely with PSQI (*ρ* = −0.56), with slightly better calibration when mask use, window-opening and stove-fuel/ventilation were modelled as dose modifiers rather than as stand-alone items. The measurement model and predictive performance are visually consistent with Figure 1 (scree) and Figure 2 (ROC). Our findings accord with large prospective cohorts linking long-term particulate exposure with stroke risk in China and elsewhere, including studies that separately examined haemorrhagic outcomes (25–27). For example, a prospective cohort in Eastern China reported higher incidence of both ischaemic and haemorrhagic stroke with increasing particulate matter exposure (25), while analyses from the Women’s Health Initiative related chronic PM exposure to stroke aetiology among older adults (7). Short-term trigger studies using case-crossover or time-series designs show near-term spikes in admissions after pollution surges (28), whereas our study targets chronic burden by integrating habitual behaviours and indoor sources; the monotonic gradient we observed across AEEQ quartiles is consistent with a dose–response pattern seen across these designs (25, 26, 28). On sleep, prior work has linked short/long sleep, irregular timing and napping with higher stroke risk and with greater blood-pressure variability (4, 11, 29, 30); we extend this literature by embedding sleep disruption within an environmental composite and demonstrating independent prediction for cerebral haemorrhage. Methodologically, most earlier studies relied on ambient mass concentrations from monitors or models (25–27) and assessed sleep using stand-alone scales; in contrast, the AEEQ captures indoor/behavioural modifiers (e.g., ventilation, fuel, mask use) that influence effective inhaled dose, which likely contributed to the stronger criterion validity with annual PM₂.₅ and the observed exposure–response. Although the AEEQ’s per-SD hazard ratio is not directly comparable with per-10-μg·m^−3^ estimates, the direction and magnitude are in the range reported for particulate pollution and small-vessel disease markers (22, 25–27, 31, 32).

The observed gradients are biologically plausible. Long-term exposure to fine particulates and traffic-related mixtures promotes endothelial dysfunction, oxidative injury and small-vessel fragility—mechanisms linked to haemorrhagic stroke risk in experimental and epidemiological literature (25, 28, 33). In parallel, irregular bedtimes, evening chronotype and fragmented sleep potentiate sympathetic surges and blood-pressure variability, diminishing nocturnal dipping and plausibly lowering the haemorrhagic threshold of penetrating arterioles (26, 27, 29, 30, 34–36). By design, the AEEQ integrates these inter-related pathways: behaviours such as mask use, window opening and stove-fuel choice are treated as conceptual modifiers of pollutant infiltration and effective inhaled dose, which likely explains the small improvement in criterion correlations and the slightly steeper exposure–response slope when the modifier specification is used. The prespecified drug-exposure interaction (stronger association among participants not using antihypertensives) is consistent with pharmacological buffering of pollutant-induced blood-pressure lability (31, 32, 37–39) and supports the mechanistic coherence of the composite. To our knowledge, few studies have jointly validated an exposure–sleep composite and then shown incremental prognostic value for haemorrhage beyond standard clinical factors; our improvement in time-dependent C-statistic and modest NRI underscores added information content relative to conventional models (26, 27).

These findings have practical implications for risk stratification and prevention. In community or primary-care settings, the AEEQ could help identify older adults whose cerebrovascular risk is under-estimated by traditional scores, prioritising air-quality mitigation (e.g., indoor filtration, ventilation practices), sleep-hygiene interventions and optimisation of blood-pressure control. At a population level, the coherent association between the composite and outcomes, together with the clear equity gradients we observed (higher scores among those with lower education, lower income, renting status and older public housing), highlights how environmental disadvantage concentrates haemorrhagic-stroke vulnerability (40–42). Linking AEEQ responses to geospatial pollution surfaces may therefore facilitate targeted allocation of mitigation resources in high-burden neighbourhoods.

Methodological features strengthen inference. The sampling frame focused on the 60–79-year risk window; exclusion of prior cerebrovascular events, heavy-industry/mining workers and heavier smokers reduced non-ambient and secondary-prevention confounding; a ≥ 5-year residential stability criterion improved the relevance of geospatial PM₂.₅ estimates; and an MMSE threshold (≥24) protected data quality. Psychometric validation followed best practice (Delphi content validation; EFA/CFA split-sample; *α* bootstrapping; two-week ICCs), and modelling choices were prespecified, including treatment of behavioural items as dose modifiers and formal testing of antihypertensive interaction. That said, limitations merit acknowledgement. First, the event count was modest (16 haemorrhages), which widens confidence intervals around effect estimates; nevertheless, the monotonic gradient across quartiles and the discrimination gain were internally consistent and robust in sensitivity analyses. Second, exposures and sleep were self-reported; any non-differential misclassification would be expected to attenuate, not inflate, hazard ratios, rendering our estimates conservative. Third, we did not include an objective noise-exposure module; because noise and air pollution co-vary spatially, residual confounding is possible and should be addressed in future iterations with integrated acoustic measures (43–46). Generalisability beyond a single municipality will require external validation in regions with different pollutant mixtures and housing stock. First, the AEEQ offers a low-burden, standardised phenotype of cumulative environmental burden that can be linked to geospatial pollution surfaces to produce equity-aware risk maps for community screening (40, 45, 46). Second, embedding the composite in electronic health records could enable pragmatic risk calculators that prompt targeted mitigation (HEPA filtration, ventilation practices), structured sleep-regularity interventions, and optimisation of antihypertensive regimens. Third, repeated AEEQ administrations would permit dynamic risk tracking and quasi-experimental evaluation of interventions (e.g., distribution of indoor filters or sleep-timing coaching) with haemorrhage and intermediate phenotypes (blood-pressure variability) as endpoints (29, 30, 45). Fourth, future iterations should integrate objective noise metrics and, where feasible, personal exposure/sleep sensors to enrich construct coverage and reduce misclassification (40, 45, 46). External validation across settings with different particle mixtures (including PM₁-dominated regions) and longer follow-up will clarify durability and generalisability of prediction (8, 24, 26, 27). The AEEQ provides a reliable, valid and implementable measure of cumulative environmental burden in older adults, and its composite score furnishes independent, dose-responsive prognostic information for cerebral haemorrhage beyond conventional risk factors. Embedding this instrument in community screening and prevention programmes may help close an actionable gap between environmental epidemiology and clinical decision-making, particularly for disadvantaged groups who bear disproportionate exposure.

## 5 Conclusion

The Anhui Environmental Exposure Questionnaire (AEEQ)—integrating chronic ambient/indoor pollution and sleep-quality metrics—showed strong psychometric performance and independently predicted 24-month risk of cerebral haemorrhage in community-dwelling older adults. The graded exposure–response and incremental discrimination beyond conventional clinical factors indicate utility for refined risk stratification and targeted prevention. Priority next steps are external validation in diverse settings and linkage with geospatial data to support equitable screening and implementation. Incorporating objective noise measures and extending follow-up will further test durability and enhance the instrument’s clinical and public-health relevance.

## Data Availability

The original contributions presented in the study are included in the article/Supplementary material, further inquiries can be directed to the corresponding authors.

## References

[1] World Health Organization. WHO global air quality guidelines: Particulate matter (PM2. 5 and PM10), ozone, nitrogen dioxide, sulfur dioxide and carbon monoxide[M]. Geneva: World Health Organization (2021). 34662007

[2] Chen IC Li CY Lu CY Huang YC Lee PC Lin MY . Psychometric properties of novel instrument for evaluating ambient air pollution health literacy in adults. PLoS One. (2023) 18:e0285001. doi: 10.1371/journal.pone.0285001, PMID: 37327221 PMC10275446

[3] Guo Y Luo C Cao F Liu J Yan J. Short-term environmental triggers of hemorrhagic stroke. Ecotoxicol Environ Saf. (2023) 265:115508. doi: 10.1016/j.ecoenv.2023.115508, PMID: 37774546

[4] Titova OE Michaëlsson K Larsson SC. Sleep duration and stroke: prospective cohort study and Mendelian randomization analysis. Stroke. (2020) 51:3279–85. doi: 10.1161/STROKEAHA.120.029902, PMID: 32895015 PMC7587241

[5] Wang Q Wu Y Wang D Lai X Tan L Zhou Q . The impacts of knowledge and attitude on behavior of antibiotic use for the common cold among the public and identifying the critical behavioral stage: based on an expanding KAP model. BMC Public Health. (2023) 23:1683. doi: 10.1186/s12889-023-16595-7, PMID: 37653367 PMC10472573

[6] Liu Z Meng H Wang X Lu W Ma X Geng Y . Interaction between ambient CO and temperature or relative humidity on the risk of stroke hospitalization. Sci Rep. (2024) 14:16740. doi: 10.1038/s41598-024-67568-8, PMID: 39033193 PMC11271280

[7] Kulick ER Eliot MN Szpiro AA Coull BA Tinker LF Eaton CB . Long-term exposure to ambient particulate matter and stroke etiology: results from the Women's Health Initiative. Environ Res. (2023) 224:115519. doi: 10.1016/j.envres.2023.115519, PMID: 36813070 PMC10074439

[8] Liu T Jiang Y Hu J Li Z Guo Y Li X . Association of ambient PM1 with hospital admission and recurrence of stroke in China. Sci Total Environ. (2022) 828:154131. doi: 10.1016/j.scitotenv.2022.154131, PMID: 35219663

[9] Wang Y Liu Q Tian Z Cheng B Guo X Wang H . Short-term effects of ambient PM1, PM2. 5, and PM10 on internal metal/metalloid profiles in older adults: a distributed lag analysis in China. Environ Int. (2023) 182:108341. doi: 10.1016/j.envint.2023.108341, PMID: 38006770

[10] Zhao X Cao J Zhou W Neophytou AM. Interactive effect of air temperature and fine particulate matter on the hospital admissions for stroke in Shenzhen, China. J Am Heart Assoc. (2025) 14:e037329. doi: 10.1161/JAHA.124.037329, PMID: 40178089 PMC12132898

[11] Zhou B Jiang C Zhang W Jin Y Zhu T Zhu F . Association of sleep duration and napping with stroke mortality in older Chinese: a 14-year prospective cohort study of the Guangzhou biobank cohort study. Sleep Med. (2023) 101:384–91. doi: 10.1016/j.sleep.2022.11.026, PMID: 36512889

[12] Degroote L DeSmet A De Bourdeaudhuij I Van Dyck D Crombez G. Content validity and methodological considerations in ecological momentary assessment studies on physical activity and sedentary behaviour: a systematic review. Int J Behav Nutr Phys Act. (2020) 17:1–13. doi: 10.1186/s12966-020-00932-9 32151251 PMC7063739

[13] Köberich S Kato NP Kugler C Strömberg A Jaarsma T. Methodological quality of studies assessing validity and reliability of the European heart failure self-care behaviour scale: a systematic review using the COSMIN methodology. Eur J Cardiovasc Nurs. (2021) 20:501–12. doi: 10.1093/eurjcn/zvab018 33864066

[14] Boogaard H Crouse DL Tanner E Mantus E van Erp AM Vedal S . Assessing adverse health effects of long-term exposure to low levels of ambient air pollution: the HEI experience and what’s next? Environ Sci Technol. (2024) 58:12767–83. doi: 10.1021/acs.est.3c09745, PMID: 38991107 PMC11270999

[15] Xu F Huang Q Yue H Feng X Xu H He C . The challenge of population aging for mitigating deaths from PM2. 5 air pollution in China. Nature. Communications. (2023) 14:5222. doi: 10.1038/s41467-023-40908-4 PMC10460422 37633954

[16] Zhao B Zhao T Yang H Fu X. The efficacy of acupressure for nausea and vomiting after laparoscopic cholecystectomy: a meta-analysis study. Surg Laparosc Endosc Percutan Tech. (2024) 34:87–93. doi: 10.1097/SLE.0000000000001196, PMID: 38095421

[17] Rajagopalan S Landrigan PJ. Pollution and the heart. N Engl J Med. (2021) 385:1881–92. doi: 10.1056/NEJMra2030281, PMID: 34758254

[18] Święczkowski M Lip GYH Kurasz A Dąbrowski EJ Tomaszuk-Kazberuk A Kamiński JW . Association between exposure to air pollution and increased ischaemic stroke incidence: a retrospective population-based cohort study (EP-PARTICLES study). Eur J Prev Cardiol. (2025) 32:276–87. doi: 10.1093/eurjpc/zwae301, PMID: 39301834

[19] Kuehu DL Fu Y Nasu M Yang H Khadka VS Deng Y. Effects of heat-induced oxidative stress and Astaxanthin on the NF-kB, NFE2L2 and PPARα transcription factors and Cytoprotective capacity in the Thymus of broilers. Curr Issues Mol Biol. (2024) 46:9215–33. doi: 10.3390/cimb46080544, PMID: 39194761 PMC11352656

[20] Zhang P Zhang H Tang J Ren Q Zhang J Chi H . The integrated single-cell analysis developed an immunogenic cell death signature to predict lung adenocarcinoma prognosis and immunotherapy. Aging (Albany NY). (2023) 15:10305–29. doi: 10.18632/aging.205077, PMID: 37796202 PMC10599752

[21] Nurmohamed NS Pereira JPB Hoogeveen RM Kroon J Kraaijenhof JM Waissi F . Targeted proteomics improves cardiovascular risk prediction in secondary prevention patients. J Am Coll Cardiol. (2022) 79:957–7. doi: 10.1016/S0735-1097(22)01948-9 PMC9020984 35139537

[22] Ma Y Hui Y Tang L Wang J Xing M Zheng L . Ambient air pollution exposure in relation to cerebral small vessel disease in Chinese population: a cranial magnetic resonance imaging-based study. Eco-Environ Health. (2025) 4:100129. doi: 10.1016/j.eehl.2024.10.004, PMID: 39925481 PMC11803214

[23] Liu R Liu J Cao Q Chu Y Chi H Zhang J . Identification of crucial genes through WGCNA in the progression of gastric cancer. J Cancer. (2024) 15:3284–96. doi: 10.7150/jca.95757, PMID: 38817876 PMC11134444

[24] Chen S Zhang Y Wei J Hao C Wu W Li Z . Risk of stroke admission after long-term exposure to PM1: evidence from a large cohort in South China. Ecotoxicol Environ Saf. (2024) 283:116720. doi: 10.1016/j.ecoenv.2024.116720, PMID: 39053181

[25] Yang L Wang M Xuan C Yu C Zhu Y Luo H . Long–term exposure to particulate matter pollution and incidence of ischemic and hemorrhagic stroke: a prospective cohort study in eastern China. Environ Pollut. (2024) 358:124446. doi: 10.1016/j.envpol.2024.124446, PMID: 38945192

[26] Kulick ER Kaufman JD Sack C. Ambient air pollution and stroke: an updated review. Stroke. (2023) 54:882–93. doi: 10.1161/STROKEAHA.122.035498, PMID: 36579640 PMC10421613

[27] Gabet S Puy L. Current trend in air pollution exposure and stroke. Curr Opin Neurol. (2025) 38:54–61. doi: 10.1097/WCO.0000000000001331, PMID: 39508397 PMC11706348

[28] Lv X Shi W Yuan K Zhang Y Cao W Li C . Hourly air pollution exposure and emergency hospital admissions for stroke: a multicenter case-crossover study. Stroke. (2023) 54:3038–45. doi: 10.1161/STROKEAHA.123.044191, PMID: 37901948

[29] Xu Y Barnes VA Harris RA Altvater M Williams C Norland K . Sleep variability, sleep irregularity, and nighttime blood pressure dipping. Hypertension. (2023) 80:2621–6. doi: 10.1161/HYPERTENSIONAHA.123.21497, PMID: 37800322 PMC10873041

[30] Kim Y Mattos MK Esquivel JH Davis EM Logan J. Sleep and blood pressure variability: a systematic literature review. Heart Lung. (2024) 68:323–36. doi: 10.1016/j.hrtlng.2024.08.016, PMID: 39217647

[31] Grande G Wu B Wu J Kalpouzos G Laukka EJ Bellander T . Long-term exposure to ambient particulate matter and structural brain changes in older adults. Stroke. (2025) 56:1816–22. doi: 10.1161/STROKEAHA.124.048096, PMID: 40353588 PMC12180693

[32] Krasnov H Sachdev K Knobel P Colicino E Yitshak-Sade M. The association between long-term exposure to PM2. 5 constituents and ischemic stroke in the new York City metropolitan area. Chemosphere. (2025) 378:144390. doi: 10.1016/j.chemosphere.2025.144390, PMID: 40203750 PMC12117512

[33] Li H Guo L Su K Li C Jiang Y Wang P . Construction and validation of TACE therapeutic efficacy by ALR score and nomogram: a large, multicenter study. J Hepatocellular Carcinoma. (2023) 10:1009–17. doi: 10.2147/JHC.S414926, PMID: 37405321 PMC10317537

[34] Chi H Huang J Yan Y Jiang C Zhang S Chen H . Unraveling the role of disulfidptosis-related Lnc RNAs in colon cancer: a prognostic indicator for immunotherapy response, chemotherapy sensitivity, and insights into cell death mechanisms. Front Mol Biosci. (2023) 10:1254232. doi: 10.3389/fmolb.2023.1254232, PMID: 37916187 PMC10617599

[35] Chen Y You Y Wei M Yang P Zhang Q Li X . Exploration of physical activity, sedentary behavior and insulin level among short sleepers. Front Endocrinol. (2024) 15:1371682. doi: 10.3389/fendo.2024.1371682, PMID: 39469577 PMC11513348

[36] Sagheer U Al-Kindi S Abohashem S Phillips CT Rana JS Bhatnagar A . Environmental pollution and cardiovascular disease: part 1 of 2: air pollution. JACC: Advances. (2024) 3:100805. doi: 10.1016/j.jacadv.2023.100805 38939391 PMC11198409

[37] Huang K Jia J Liang F Li J Niu X Yang X . Fine particulate matter exposure, genetic susceptibility, and the risk of incident stroke: a prospective cohort study. Stroke. (2024) 55:92–100. doi: 10.1161/STROKEAHA.123.043812, PMID: 38018834 PMC11831602

[38] Qiang C Qi Z Yi Q. Mechanisms of p2x7 receptor involvement in pain regulation: a literature review. Acta Med Mediterr. (2022) 38:1187–94.

[39] You Y Chen Y You Y Zhang Q Cao Q. Evolutionary game analysis of artificial intelligence such as the generative pre-trained transformer in future education. Sustainability. (2023) 15:9355. doi: 10.3390/su15129355

[40] Poulsen AH Sørensen M Hvidtfeldt UA Christensen JH Brandt J Frohn LM . Concomitant exposure to air pollution, green space, and noise and risk of stroke: a cohort study from Denmark. Lancet Reg Health–Europe. (2023):31. doi: 10.1016/j.lanepe.2023.100655 PMC10230828 37265507

[41] Song G Peng G Zhang J Song B Yang J Xie X . Uncovering the potential role of oxidative stress in the development of periodontitis and establishing a stable diagnostic model via combining single-cell and machine learning analysis. Front Immunol. (2023) 14:1181467. doi: 10.3389/fimmu.2023.1181467, PMID: 37475857 PMC10355807

[42] Marfella R Gualdiero P Siniscalchi M Carusone C Verza M Marzano S . Morning blood pressure peak, QT intervals, and sympathetic activity in hypertensive patients. Hypertension. (2003) 41:237–43. doi: 10.1161/01.HYP.0000050651.96345.0E, PMID: 12574088

[43] Su K Wang F Li X Chi H Zhang J He K . Effect of external beam radiation therapy versus transcatheter arterial chemoembolization for non-diffuse hepatocellular carcinoma (≥ 5 cm): a multicenter experience over a ten-year period. Front Immunol. (2023) 14:1265959. doi: 10.3389/fimmu.2023.1265959, PMID: 37818373 PMC10560878

[44] Ahn S Howie-Esquivel J Davis EM Chung ML Lobo JM Logan JG. Association of disrupted sleep with 24-hour blood pressure variability in caregivers. Heart Lung. (2023) 60:45–51. doi: 10.1016/j.hrtlng.2023.02.024, PMID: 36905754

[45] Ding R Ren X Sun Q Yang M Wang Y Sun Z . Air pollution and stroke: an emerging challenge from cardio-cerebrovascular multimorbidity. J Am Heart Assoc. (2025) 14:e041848. doi: 10.1161/JAHA.124.041848, PMID: 40576036 PMC12449924

[46] Blaustein JR Quisel MJ Hamburg NM Wittkopp S. Environmental impacts on cardiovascular health and biology: an overview. Circ Res. (2024) 134:1048–60. doi: 10.1161/CIRCRESAHA.123.323613, PMID: 38662864 PMC11058466

